# A case of otogenic septic arthritis of the knee

**DOI:** 10.1093/jscr/rjad682

**Published:** 2023-12-18

**Authors:** Leyla Ozbek, Jonathon Kyriakides, Ajay Asokan

**Affiliations:** Otolaryngology Department, University College Hospital, 235 Euston Road, London NW1 2BU, United Kingdom; Trauma and Orthopaedics Department, Barnet Hospital, Wellhouse Lane, London EN5 3DJ, United Kingdom; Trauma and Orthopaedics Department, Barnet Hospital, Wellhouse Lane, London EN5 3DJ, United Kingdom

**Keywords:** septic arthritis, otitis media, suppurative, Streptococcus pyogenes, suppurative arthritis

## Abstract

Septic arthritis is a serious condition resulting in rapid destruction of articular cartilage and potential sepsis. Bacterial invasion of a joint occurs most commonly as a result of haematogenous spread from a distant infection. However, an otogenic source of this transient bacteraemia and resultant septic arthritis has not yet been reported in the literature. We report a case of acute septic arthritis of the knee with *Streptococcus pyogenes*, secondary to acute otitis media of the ear.

## Introduction

Acute septic arthritis (SA) is a consequence of bacterial invasion into a joint, resulting in rapid destruction of articular cartilage and sepsis. It is an uncommon cause of atraumatic monoarticular pain and swelling, with an incidence of 5–10 per 100 000 in the United Kingdom [1]. This condition is associated with significant morbidity and mortality; prompt diagnosis and management is therefore necessary to prevent irreversible joint damage and sepsis [2].

SA is most frequently a result of haematogenous spread following an episode of bacteraemia [3]. Other causes of SA include direct inoculation from either trauma or medical intervention, or contiguous spread from an adjacent osteomyelitis, abscess, or cellulitis [4]. *Staphylococcus aureus* is the most common organism, followed by *Streptococcus* species [5]. Gramme-negative organisms, most frequently *Pseudomonas*, account for ~15% of SA [4].

We present an unusual case of SA of the knee following an episode of acute otitis media (AOM). We propose haematogenous spread from a distant site, and use this case to highlight a rare but potential source of inoculation.

## Case report

A male in his early 40s patient presented to the emergency department (ED) with a 3-day history of atraumatic pain and swelling of the left knee. One week prior, the patient reported coryzal symptoms with left otalgia and discharge. He was reviewed by a general practitioner and prescribed a course of antibiotics for AOM, which the patient did not commence. He had no medical history of note, and denied previous trauma, intervention, or surgery to the knee.

In the ED, he had a temperature of 38.9°C and was tachycardic, with a blood pressure of 105/65 mmHg. His left knee was erythematous and warm to touch, with a large joint effusion and severely limited active and passive range of movement.

Initial blood tests revealed a haemoglobin of 156 g/L, white cell count (WCC) 28.63 ×10^9^/L, neutrophil count 26.40 ×10^9^/L, C-reactive protein (CRP) level 531 mg/dL, and lactate 3.2 mmol/L. Plain radiographs of the knee showed a large joint effusion, with no evidence of bony destruction. The knee was aspirated, and 40 ml of frank pus was sent for microscopy and culture. Exudate from the ear was also sent for analysis.

The patient underwent arthroscopic washout of the knee the same evening. Intra-operatively he was found to have further frank pus, and significant synovitis. No acute articular damage was seen. Intra-operative fluid and synovial tissue samples were taken, and the knee was irrigated with 6 l of normal saline.

During induction of anaesthesia, the left ear discharged pus, which was sent for culture. The ear, nose, and throat teams were consulted, and otoscopy of the left ear revealed pus within an erythematous external auditory canal and a small tympanic membrane perforation; the team advised ciprofloxacin ear drops.

Following initial washout, the patient improved clinically, although movement of the knee remained restricted. After initial improvement, inflammatory markers remained static; a WCC of 16.65 ×10^9^/L and CRP of 239 mg/dL prompted two further arthroscopic washouts.

**Figure 1:**
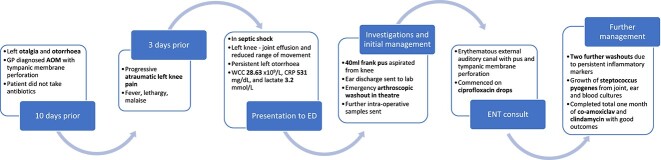
Timeline of events.

Intra-operative samples, joint aspirate, and blood cultures grew *Streptococcus pyogenes*. The swab of ear discharge revealed growth of *S. pyogenes, S. aureus*, and *Haemophilus influenzae*. The patient was commenced on a targeted regimen of intravenous co-amoxiclav and clindamycin. He continued these antibiotics for a total of 1 month, stepping down to an oral regimen after 2 weeks. Prior to discharge, his inflammatory markers normalized and the patient was able to weight-bear, with normal range of movement in the knee. See Fig. 1 for a timeline of events.

## Discussion

AOM is acute inflammation of the middle ear cleft, which can be bacterial or viral. Our patient was found to suffer from a subtype, acute suppurative otitis media, in which the tympanic membrane perforates in 5% of cases [7]. AOM is common in infants; more than 80% of children experience at least one episode [8]; however, it is much less commonly encountered in adults, with an incidence of 0.25% [9]. The risk factor profile is similar across all ages, and includes smoking (active or passive), eustachian tube dysfunction, upper respiratory tract infection, and sinusitis [7].

AOM is well documented to lead to a number of local complications including mastoiditis, meningitis, and abscesses [7]; however, haematogenic spread to distant sites is exceedingly rare; otogenic SA, as seen in our patient, is only reported in the literature to occur at the temporo-mandibular joint in the paediatric population [10]. Large joint SA from an otogenic cause has not yet been reported in the literature.

*Streptococcus* species is the second most prevalent species in the pathogenesis of SA. It most frequently affects the paediatric population [5], with a varying age of onset of 3 months to 7 years [11]. In the adult population, isolated cases of SA because of *S. pyogenes* are reported in the literature [12–14], although none of these were otogenic in origin.

Our patient reported a preceding history of coryzal symptoms with ear discharge for 1 week prior to developing knee pain and swelling. All three cultures derived from the joint, ear, and blood grew *S. pyogenes*, and thus we conclude that his SA was otogenic in nature. No causal risk factors for developing a virulent strain with haematogenic spread were identified, but the patient’s non-adherence to initial antibiotic therapy for his AOM will likely have exacerbated the infection. Nonetheless, the patient was given prompt diagnosis and treatment in the form of surgical washout and targeted antibiotics, thus acute joint destruction was not seen. The risk of long-term joint dysfunction in SA is cited as 30% [6, 15], which cannot yet be evaluated in this patient.

## Conclusion

This case report presents a unique case of acute supportive otitis media leading to SA of the knee, which is previously unreported in the literature. *Streptococcus pyogenes* is a common organism in otogenic infection, but can be particularly virulent in SA, thus prompt diagnosis and treatment is required, and this case report brings to the readers’ attention a rare but serious complication of AOM.
